# Treatment of Class III malocclusion using Hybrid Hyrax, Face Mask and Alt-RAMEC Protocol: A Case Report in a Latin-American patient

**DOI:** 10.4317/jced.55939

**Published:** 2019-07-01

**Authors:** Robinsón-Andrés Castrillón-Marín, Diana-María Barbosa-Liz, Carlos-Martin Ardila

**Affiliations:** 1 Orthodontist. Professor School of Dentistry. Universidad de Antioquia. Medellin. Colombia; 2 Orthodontist. Titular Professor. Gionorto Research Group. Department of Orthodontics. School of Dentistry. Universidad de Antioquia. Medellin. Colombia; 3 Periodontist. Ph.D in Epidemiology; Biomedical Stomatology Research Group, Universidad de Antioquia, Medellín, Colombia

## Abstract

The management of Class III malocclusion is one of the greatest challenges of orthodontics. Current treatments offer the possibility of using direct skeletal anchorage to improve clinical outcomes. This case shows the results of using a Hyrax hybrid palatal anchorage, Alt-RAMEC (Alternate Rapid Maxillary Expansion and Contraction protocol) and a facemask to treat a maxillary hypoplasia Class III malocclusion in a Latin-American patient. The appliance design and the protocol used are widely described. Clinical and cephalometric results suggest that it is a good treatment option for this Latino patient, with moderate malocclusion and limitations in the dental anchorage.

** Key words:**ALT-RAMEC, Angle Class III, malocclusion, maxillary expansion, mini-screws, orthodontic anchorage, TADs.

## Introduction

The objective of the treatment of growing patients with occlusal and skeletal Class III is to achieve a positive overjet through a combination of skeletal and dentoalveolar changes (1). Consequently, a rapid maxillary expansion (RME) with a facial mask (FM) has been commonly used (2). However, there are certain disadvantages associated to a dental anchorage device, which can cause a loss of space in the maxillary arch and prohibits the application of orthopedic force directly on the bone, limiting maxillary advancement (3).

Anchorage protocols have been developed to avoid the undesirable effects of dental anchorage, including dental implants, surgical mini-plates and ankylosed teeth (4). To minimize the invasiveness of these procedures, the Hybrid Hyrax was implemented (5), using mini-implants in the anterior region of the palate as sagittal bone support to prevent mesial migration of the maxillary dentition and to apply orthopedic force directly on the bone. Also, it was suggested the use of a protocol of alternating expansion and contraction of the palatal suture through Hyrax to potentiate the effects of maxillary protraction with FM; although most studies have shown favorable results (6), there is still controversy around them. A study reported that the Alt-RAMEC/FM protocol produced a more effective advancement of the maxilla and greater inter-maxillary changes (7). Also, a systematic review reported a positive influence on the maxillary protraction using the Alt-RAMEC protocol and reduced dental side effects on upper incisor angulation (8).

Some authors have reported different facial, dental, skeletal and cephalometric characteristics between different races and ethnicities; for example, Hispanics patients have lower mandibular clockwise rotation than Japanese patients, and Latino patients showed more vertical incisors (9). Johe *et al.* (10) found that Hispanic patients have some tends to have a superior mandibular excess.

As can be seen, the clinical characteristics of patients in different ethnic groups may differ; consequently, the response to the treatment could be an interesting aspect to be investigated. For our knowledge, there is no scientific literature that reports the results of this protocol in Latin-American patients. The purpose of this report is to illustrate the use of a Hyrax supported by two mini-implants on the palate, RME using the Alt-RAMEC protocol, and maxillary FM before the pubertal growth spurt in a Latin-American patient.

## Case Report

-Diagnosis 

A 10-year-old Latino Colombian female was attended with a main complaint: “My lower teeth lean farther back as my jaw grows”. She presented good general health and no systemic or congenital disease. She had received RME through a Hyrax appliance six months earlier.

**Figure 1:**
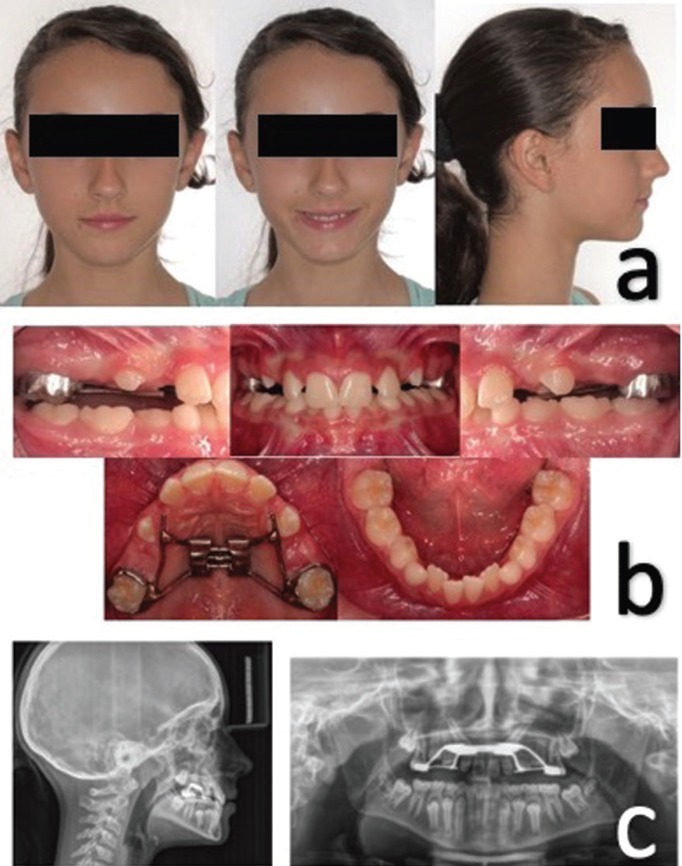
a) Extra oral clinical photographs pre-treatment. b) Intraoral photographs of the pre-treatment. c) Pre-treatment lateral and panoramic X-ray.

She presented a straight profile, infraorbital, malar and paranasal hypoplasia, obtuse nasolabial angle and competent lips (Fig. 1a). Panoramic, cephalic lateral X-rays and dental cast records were taken. The cephalometric analysis confirmed the skeletal Class III relationship (Wits Appraisal -4mm), maxillary retrognathism (SNA 78°), maxillary micrognathism (Co-A 83mm) and retro-inclined lower incisors ( Table 1), showing compensation of the Class III malocclusion. Intraorally, the patient had an anterior deep bite (71.4%), 2 mm overjet and class I molar relationship (Fig. 1b). She also presented a poor prognosis for the eruption of 13 and 23 (Fig. 1c) and she was in stage CS1, according to the analysis of vertebral maturation (6). The patient was in a final mixed dentition, which limited the possibilities of dental anchorage due to the physiological mobility of the deciduous molars and the radicular immaturity of the present premolars.

**Table 1:**
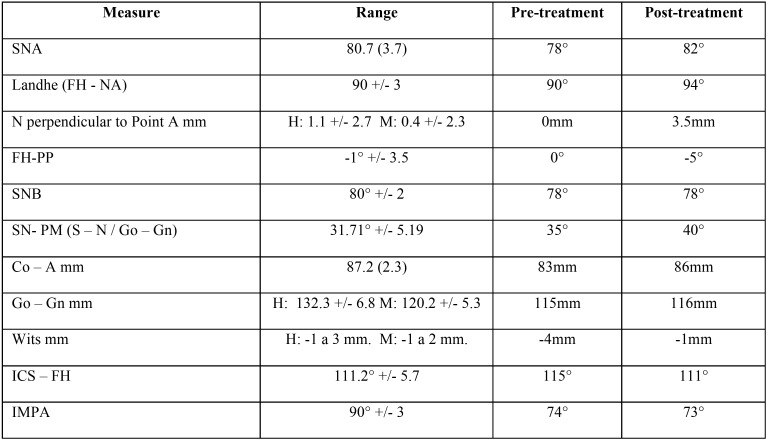
Cephalometric measures pre and post-treatment.

The treatment objectives were to achieve a maxillary advancement to correct midface hypoplasia, enlarge the upper arch, improve the profile, promote mandibular rotation down and backward to correct the deep bite, and promote the spontaneous eruption of upper canines. The prognosis for the maxillary advancement was not very good since the age of the patient showed that the maxilla was already finishing its growth.

-Treatment

**Figure 2:**
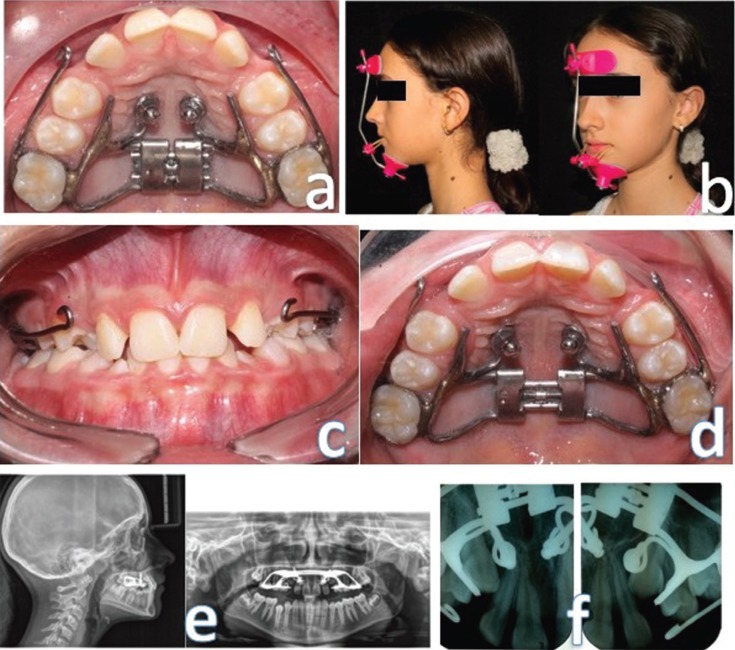
a) Hybrid Hyrax Installed. b) Maxillary Protraction with Face Mask. c) Frontal intraoral image after 9 weeks of Alt-RAMEC. d) Palatal intraoral image after 9 weeks of Alt-RAMEC. e) Lateral and panoramic X-ray after treatment. f) Periapical X-ray of the upper right and left canine after treatment.

For rapid maxillary expansion, a Hyrax was made with an 11-mm expansion screw adapted to bands on 16 and 26, buccal arms that extend to the canine area, and two 0.048 stainless steel rings at the palatal level for the insertion of mini-implants. Under local anesthesia, two 3M Unitek A1 mini-implants (2 mm diameter, 10 mm length) were inserted, adjacent to the middle suture, and at the level of the second and third palatal wrinkles (Fig. 2a). Then, the screw was activated with a 90° turn twice a day for daily 0.5 mm activation, following a protocol described (11). After one week of expansion, the screw was activated for compression in the next week, according to the ALT-RAMEC protocol; this sequence was continued for 7-9 weeks. Concomitant, an orthopedic force of maxillary protraction of 400gm per side with intermaxillary elastics connected to the FM was activated with 25 degrees of inclination, with a frequency of use of 10 to 14 hours daily (Fig. 2b). The adherence of the patient to the treatment was good and none adverse events occurred.

Written consent of the parents and of the patient according to ethical principles was signed.

## Results

Figures 2c, d, e show the results obtained after 10 months of daily use of FM. Rapid maxillary expansion was performed with the Alt-RAMEC protocol for 9 weeks, ending in the expansion stage, which resulted in a better conformation of the upper arch and an increase in the perimeter of the arch.

The cephalometric tracings show an anterior displacement of the point A of 3.5mm (┴ N – point A) and 4° (SNA, Landhe), as well as a counterclockwise rotation of 5° on the palatal plane (Fig. 2e). The inter-maxillary relationship improved as the ANB angle went from 0° to + 4°, the Wits appraisal changed 3mm, and a 5° mandibular clockwise rotation improved the patient’s overbite ( Table 1). Additionally, there was no vestibular inclination of the upper incisors or retro-inclination of the lower incisors, which is a typical outcome of the use of the dentoalveolar FM, due to the skeletal anchorage used. In addition, control radiographs of the upper canines were taken, and an improvement in their eruption trajectories was evidenced (Fig. 2f).

**Figure 3:**
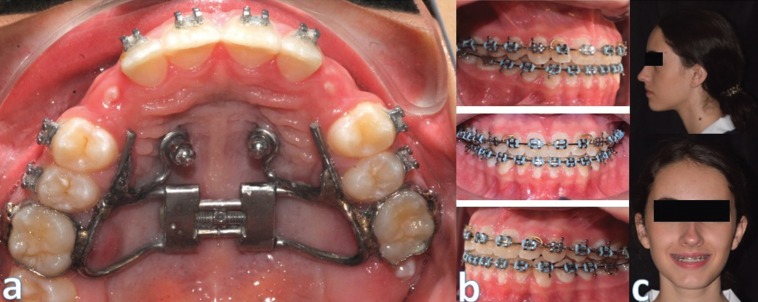
a) Improve in canines’ eruption pathway. b) Intra oral follow up after 18 months. c) Extra oral follow up after 18 months.

Figure 3a shows no mesial displacement of the posterior segments, assuring the necessary space for the eruption of the upper canines and demonstrating the efficiency of the anchorage. Finally, regarding the facial impact of the treatment, the patient presented a better expression of the middle third and a greater projection of the subnasal tissue and the upper lip. Figures 3b and 3c present the follow-up after 18 months.

## Discussion

The success of treatment in patients who are developing a Class III malocclusion depends on individual growth and when the therapeutic intervention is performed. Here, the decision to perform an early treatment or wait for growing was difficult; the facial features did not show an important skeletal compromise because the discrepancy was camouflaged by dentoalveolar compensations. Therefore, it is necessary to provide a precise skeletal diagnosis that allows a correct therapeutic decision that improves the intermaxillary relationship and promotes adequate growth.

Currently, in the treatment of young patients with skeletal and occlusal Class III, concomitant maxillary rapid expansion with FM has been used (5). Maxillary protraction with dental anchorage includes adverse effects, such as the loss of arch perimeter length by the mesialization of posterior segments and periodontal defects caused by the distribution of the expansive force through the dental anchor units (12). Various authors (6,11) have developed a protocol for maxillary protraction with bone anchorage, where the side effects of conventional face mask are mitigated, and the degree of skeletal correction is greater.

In this patient, a significant skeletal improvement was achieved at the maxillary in the sagittal plane, confirmed in the angle SNA (4°) and Wits (3mm). These values are higher than those found previously (12), demonstrating that skeletal anchorage provides a greater advance than the dental anchorage, concordant with the results obtained recently (13). Additionally, ALT-RAMEC protocol has shown improved outcomes for maxillary protraction. This case combined the advantages of implant anchorage and ALT-RAMEC protocol to improve maxillary protraction.

Here, there was a 5° counterclockwise rotation of the palatal plane and a slight increase in mandibular downward and backward rotation, despite the use of the modification by Keles *et al.* (14), where the force vector was applied 30° below the occlusal plane to compensate for the application of the force lower to the maxillary resistance center. However, a long-term study suggested that the palatal plane inclination following maxillary protraction returns to its normal value (15), then the radiographic follow-up will be important to evaluate the correction in this patient.

One of the most important factors in the treatment of Class III malocclusions is the time at which the intervention is initiated. The patient was treated before the pubertal growth spurt, stage CS1 according to Baccetti *et al.* (15), then the circummaxillary sutures could be stimulated for maxillary expansion and protraction aided using bone anchorage to promote greater skeletal changes.

The canine eruption pattern improved with the treatment (Fig. 2c). Baccetti *et al.* (15) evidenced the relationship between palatal expansion and the change in canine eruption path, thus this expansion could increase the space available for the eruption of the canine. Additionally, upper incisors were already spaced, and the consolidation of upper incisor also contributed for the same.

The limitations of this report are related to the presentation of only one case and to the short-range assessment of the treatment result. Experimental studies will be necessary to evaluate the efficacy of the modified Alt-RAMEC protocol.

Although there are differences in the clinical and cephalometric characteristics in different populations, the combination of Hyrax Hybrid, facemask, and Alt-RAMEC protocol, presented in this report, was efficient for the management of class III malocclusion in a Latin-American patient.
